# Initiating a New Era of Cardiovascular Diagnosis and Therapy in Acute Aortic Syndromes: The Mainz–Essen Experience (Part I)—Imaging and Biomarkers

**DOI:** 10.1055/s-0041-1730295

**Published:** 2021-11-08

**Authors:** Eduardo Bossone, Riccardo Gorla, Brigida Ranieri, Valentina Russo, Heinz Jakob, Raimund Erbel

**Affiliations:** 1 Division of Cardiology, A. Cardarelli Hospital, Naples, Italy; 2 Division of Cardiology, Scientific Institute for Research, Hospitalization and Healthcare Policlinico San Donato, San Donato Milanese, Milan, Italy; 3 Department of Imaging, Scientific Institute for Research, Hospitalization and Healthcare SDN, Naples, Italy; 4 Department of Advanced Biomedical Sciences, Federico II University of Naples, Naples, Italy; 5 Department of Thoracic and Cardiovascular Surgery, West German Heart and Vascular Center, University of Duisburg-Essen, University Hospital Essen, Essen, Germany; 6 Institute for Medical Informatics, Biometry and Epidemiology (IMIBE), University of Duisburg-Essen, University Hospital Essen, Essen, Germany

**Keywords:** acute aortic syndromes, Mainz–Essen, acute aortic dissection, hybrid operating room, heart team concept

## Abstract

Acute aortic syndromes (AAS) encompass a group of life-threatening medical conditions (acute aortic dissection [AAD], intramural hematoma, and penetrating aortic ulcer) with a common pathophysiological pathway. Due to overlapping symptoms and signs with other cardiovascular emergencies, the diagnosis remains challenging resulting in time delays and related increased in-hospital and long-term morbidity and mortality. The Cardiovascular Department of Johannes Gutenberg University in Mainz at West-German Heart Centre in Essen (Germany) first described (in 1984) AAD by transesophageal echocardiography, AAD diagnostic features, and furtherly explored the implementation of “invasive” imaging techniques, namely, intravascular ultrasound and intraluminal phased-array imaging. Furthermore, pioneer studies were undertaken on the biomarker and imaging interplay, namely, D-dimer and F-fluorodeoxyglucose positron emission tomography/computed tomography. We discuss the unique 35-year-long Mainz–Essen experience on the diagnostic and prognostic role of serological and imaging biomarkers in AAS.

## Introduction

Acute aortic syndromes (AAS) encompass a group of life-threatening medical conditions with a common pathophysiological pathway (i.e., breakdown of intima and media) that leads to different clinical scenarios, including acute aortic dissection (AAD), intramural hematoma (IMH), penetrating aortic ulcer (PAU), and traumatic aortic injury ( Tables 1 and 2 ). 1 2 3 4 5 6 7 8

**Table 1:** Acute aortic syndromes: definition and incidence

AAS	Definition 1	Incidence a	Remarks 1
Aortic dissection (85–95%)	Disruption of the medial layer provoked by intramural bleeding, resulting in separation of the aortic wall layers and subsequent formation of a true lumen and a false lumen (FL) with or without communication	2.6–6 2 3 4	• The real incidence is difficult to define due to pre-hospital mortality and/or missing diagnosis • The incidence is higher in men and increases with age. • *Types* : 5 67% Type A 33% Type B
Intramural hematoma (10–25%)	Presence of hematoma in the media of the aortic wall in the absence of an FL and intimal tear	∼1.2 2	• Mainly in older patients • *Types* : 10–30% Type A 60–70% Type B •30–40% of Type A IMH evolved into AD
Penetrating aortic ulcer (2–7%)	Ulceration of an aortic atherosclerotic plaque penetrating through the internal elastic lamina into the media	∼2.1 2	• Often multiple and different in size and depth. • More frequent in older age, male gender and in patients with atherosclerotic cardiovascular disease. • *Types* 6 : Rare in ascending aorta ∼17.5% aortic arch ∼68% descending aorta ∼14% thoracoabdominal transition

Abbreviation: AAS, acute aortic syndromes.

Note: Data from references. 1 2 3 4 5 6

a All data are per 100,000 person-years.

**Table 2:** Classification systems of acute aortic syndromes 1

Classification	Types/Categories	Description
Stanford	Type A	All dissections involving the ascending aorta irrespective of the site of tear
	Type B	All dissections that do not involve the ascending aorta; note that involvement of the aortic arch without involvement of the ascending aorta in the Stanford classification is labeled as Type B
DeBakey	Category I	Dissection tear in the ascending aorta propagating distally to include at least the aortic arch and typically the descending aorta
	Category II	Dissection tear only in the ascending aorta
	Category III	Dissection tear in the descending aorta propagating most often distally
	Category IIIa	Dissection tear only in the descending thoracic aorta
	Category IIIb	Tear extending below the diaphragm
Svensson	Class I	Classical dissection with true and false lumen
	Class II	Intramural hematoma or hemorrhage
	Class III	Subtle dissection without hematoma
	Class IV	Penetrating atherosclerotic aortic ulcer
	Class V	Iatrogenic or traumatic dissection
Time course: from symptom onset to presentation Erbel et al	Acute	<14 days
	Subacute	15–90 days
	Chronic	>90 days

Note: Modified from references. 1 7 8

Due to overlapping symptoms and signs with other cardiovascular emergencies (namely, acute coronary syndromes, pulmonary embolism, and stroke), the diagnosis remains challenging resulting in time delays and related increased in-hospital and long-term morbidity and mortality. Thus, a high clinical index of suspicion is needed from the caring physician (team) to make a timely diagnosis and proceed to the appropriate diagnostic tests (biomarkers and imaging) and therapeutic interventions. During the last decades, clinical and laboratory (biomarkers and genetic) and imaging data derived by single centers, as well as multiple registries and population-based studies along with consensus statements/guidelines, developed by American and European Specialty Societies, have provided key insights for designing optimal diagnostic and therapeutic pathways. 1 9 In Europe, the Cardiovascular Department of Johannes Gutenberg University in Mainz, Germany, first described AAD by transesophageal echocardiography (TEE) in 1984 10 and furtherly investigated the role of invasive imaging (mainly TEE and intravascular ultrasound [IVUS]) versus computed tomography (CT) to rapidly confirm AAS without delaying treatment. Furthermore, pioneer studies were undertaken on the diagnostic and prognostic role of biomarker and imaging interplay, namely, D-dimer and F-fluorodeoxyglucose (FDG) positron emission tomography (PET)/CT. Twenty years later, the West-German Heart Centre in Essen, Germany, developed the concept of “hybrid operating room (HOR)” and established it in 2003 aiming at an integrated invasive diagnostic assessment and treatment in AAS patients, and becoming, over years, a center of excellence with great expertise in this field. We report and discus this unique 35-year-long Mainz–Essen experience in the field of diagnosis (part I) and management (part II) of AAS ( Tables 3 and 4 ; Supplementary Box S1 , including Supplementary Fig. S1 [available in the online version]). 11 12 13 14 15 16 17 18 19 20 21 22 23 24 25 26 27 28 29 30

**Table 3:** Studies investigating the role of different imaging modalities in patients with acute aortic syndromes (AAS)

Study (year)	Study type	Sample size ( *n* )	Mean age (y) Gender (male [M], female [F])	Imaging modality	Mean follow-up	Main findings
Erbel et al (1987) 11	Prospective single center	21 aortic dissection (AD) patients	60.14 9 M, 12 F	Transthoracic echocardiography/transesophageal echocardiography (TTE/TEE)	36 months	TTE+TEE identified correctly the degree of AD in all patients
Erbel et al (1989) 12	Prospective multicenter	164 patients (82 with acute aortic dissection [AAD])	56.0 Gender = not available (NA)	TTE/TEE versus computed tomography (CT) versus aortography	NA	*TTE + TEE* : Sensitivity = 99% Specificity = 98% *CT* : Sensitivity = 83% Specificity = 100% *Aortography* Sensitivity 88% Specificity 94%
Mohr-Kahaly et al (1989) 13	Retrospective single center	18 AAD patients	51.6 13 M, 5 F	TEE	15 months	TEE as ambulatory tool to detect AAD-related complications leading to reintervention and to monitor the healing process
Erbel et al (1993) 14	Prospective multicenter	168 AAD patients	Range: 23–84 124 M, 44 F	TEE	10 months	Characterization of AAD morphology at TEE and relation to short- and long-term prognosis Reoperation higher in Type-III DeBakey AAD with communications and antegrade dissection Survival higher for Type-III noncommunicating and III communicating and retrograde dissection limited to descending aorta Poor prognostic factors: fluid extravasation and an open false lumen with high communication Good prognostic factor: thrombus formation in the false lumen
Mohr-Kahaly et al (1994) 15	Prospective single center	114 patients (15 intramural hematoma [IMH] patients)	70.0 12 M, 3 F	TEE	11 months	Identification of the echocardiographic features of IMH TEE is a useful tool for detection of IMH and monitoring its evolution
Epperlein et al (1994) 16	Retrospective double center	139 AAD patients	60.5 ± 15.7 96 M, 43 F	TEE	NA	TEE detects AAD, underlying pathologies of aorta/aortic valve as well as predisposing aortic valve morphologies
Weintraub et al (1994) 18	Retrospective multicenter	28 patients (23 with AD)	Range: 28–79 12 M, 11 F	Intravascular ultrasound (IVUS)	NA	IVUS is easy and safe and allows to visualize the degree of lumen compression, presence of clots in the false lumen, periaortic hematoma, and involvement of aortic branches
Bartel et al (2007) 17	Prospective single center	23 Type B AD patients	63.0 ± 13.0 18 M, 5 F	Intraluminal phased-array imaging (IPAI) versus TEE versus IVUS	NA	IPAI is superior to IVUS and TEE in detecting communications between true and false lumen IPAI is highly useful as a guiding tool for emergency intimal flap fenestration
Kuehl et al (2008) 23	Prospective single center	33 AAS patients	67.0 28 M, 5 F	positron emission tomography (PET)/CT	224 (standard deviation = 195)	Vessel wall inflammation is a risk factor for disease progression
Jánosi et al (2015) 19	Retrospective single center	57 AAS patients	65.3 ± 16.0 31 M, 26 F	IVUS versus CT	NA	IVUS is a reliable tool for measuring aortic diameter, especially in the descending part of the aorta to choose a correctly sized stent–graft
Gorla et al (2015) 24	Retrospective single center	60 Type B AAS patients	68.8 ± 11.7 42 M, 18 F	PET/CT	3 years	PET positivity is associated with all-cause mortality at 3-year follow-up Combination of PET positivity and D-dimer levels has the best discriminant value of major aortic events
Lortz et al (2018) 20	Retrospective single center	45 Type B AD patients undergoing thoracic endovascular aortic repair (TEVAR)	66.9 ± 10.0 10 M, 10 F (IVUS: *n* = 20) 62.3 ± 14.2 10 M, 15 F (CT: *n* = 25)	IVUS versus CT	24.4 ± 22.8 months	IVUS-assisted sizing yielded a greater increase in true lumen and reduction of the false lumen and total aortic diameter, compared with CT-assisted sizing
Lortz J et al (2018) 21	Retrospective single center	115 AAS patients (83 elective patients, 32 emergent patients)	66.1 ± 12.6 47 M, 36 F (83 elective) 59.4 ± 16.7 21 M, 62 F (32 emergent)	IVUS versus CT	NA	IVUS may be useful in emergent situations to allow correct aortic sizing for stent-graft selection after adjustment of patient's hemodynamics CT-assisted sizing might lead to underestimated aortic diameters in case of impaired hemodynamics
Rylski et al (2018) 22	Retrospective multicenter	802 AAD patients (25 Type B AAD eligible patients)	Median = 60.3 15 M, 10 F	CT	NA (median time = 12 months)	The pre-AAD aortic diameter of the proximal thoracic descending aorta closely resembled the maximum true-lumen diameter

**Table 4:** Studies addressing the role of biomarkers in patients with acute aortic syndromes (AAS)

Study (year)	Study type	Sample size ( *n* )	Mean age (y) Gender (male [M], female [F])	Biomarkers	Mean follow-up	Main findings
Eggebrecht et al (2004) 25	Retrospective multicenter	64 patients with chest pain (16 acute aortic dissection [AAD] patients)	65.2 ± 15.2 11 M, 5 F	D-dimer (D-d), white blood cell count (WBC), C-reactive protein (CRP), fibrinogen (FBG)	Not available (NA)	D-d is highly elevated in AAD and pulmonary embolism, compared with other causes of chest pain A systemic inflammatory reaction is evident in AAD patients
Eggebrecht et al (2008) 26	Retrospective single center	103 patients undergoing thoracic endovascular aortic repair (TEVAR)	64.5 ± 11.2 70 M, 33 F	D-d, WBC, CRP, FBG	26.1 ± 23.9 months	All biomarkers significantly increase after TEVAR with different kinetics, particularly in patients with AAS and those receiving >1 stent-graft Patients with highest postoperative D-d level had poor survival during follow-up
Gorla et al (2016) 29	Retrospective single center	133 Type B AAS patients undergoing TEVAR	71.3 ± 12.0 86 M, 47 F	D-d, WBC, CRP, FBG, interleukin (IL)-6	4.0 ± 2.9 years	The biomarkers increase after TEVAR is more intense in those who develop post-implantation syndrome (PIS) IL-6 was PIS specific PIS predicted major aortic events during follow-up
Gorla et al (2017) 27	Retrospective single center	231 AAS patients (159 AAD)	67.3 ± 13.0 154 M, 77 F ( *n* = 231 AAS patients) 64.6 ± 13.6 110 M, 49 F ( *n* = 159 AAD patients)	D-d	1,055 ± 1,186 days ( *n* = 231 AAS patients) 1,055 ± 1,212 days ( *n* = 159 AAD patients)	D-d was a reliable diagnostic marker for AAD and intramural hematoma, but not for penetrating aortic ulcer Persistently high D-d levels during hospitalization were independent predictors of in-hospital mortality
Gorla et al (2017) 28	Retrospective single center	376 patients with chest pain (85 AAS patients)	63.1 ± 12.1 231 M, 145 F ( *n* = 376 chest pain patients) 65.6 ± 12.0 56 M, 29 F ( *n* = 85 AAS patients)	D-d	NA	A “high probability” aortic dissection detection score detected AAS with good specificity A ‘low probability’ score combined with negative D-d safely and efficiently ruled out AAS with a low failure rate
Gorla et al (2017) 30	Retrospective single center	144 Type B AAS patients undergoing TEVAR (93 AAD patients)	69.1 89 M, 55 F	Hemoglobin (Hb), creatinine	2.6 years	Preoperative Hb and postoperative Hb drop were significant risk factors for acute kidney injury Postoperative Hb drop and postoperative Hb levels predicted in-hospital mortality

## Role of Different Imaging Modalities in Acute Aortic Syndromes

Diagnostic imaging represents an essential step in the diagnostic and prognostic pathways of AAS. In the emergency scenario, CT is the most commonly used imaging modality (first choice in two-thirds of patients) followed by TEE. Magnetic resonance imaging (MRI) for its intrinsic characteristics is much less implemented being more suitable for follow-up. Being invasive, retrograde aortography (historic gold standard) is performed only when coronary angiography and/or endovascular interventions are planned. Each patient usually undergoes more than one imaging modality before definitive diagnosis is made and any appropriate treatment is undertaken. Table 5 describes the diagnostic value, advantages, and disadvantages of each technique. 1

**Table 5:** Diagnostic value and advantages/disadvantages of imaging modalities for acute aortic syndromes

Diagnostic value/Advantages/Disadvantages	Transthoracic echocardiography	Transesophageal echocardiography	Computed tomography	Magnetic resonance imaging
*Diagnostic value* :				
Ascending aortic dissection	+ +	+ + +	+ + +	+ + +
Aortic arch dissection	+	+	+ + +	+ + +
Descending aortic dissection	+	+ + +	+ + +	+ + +
Size	+ +	+ + +	+ + +	+ + +
Mural thrombus	+	+ + +	+ + +	+ + +
Intramural hematoma	+	+ + +	+ +	+ + +
Penetrating aortic ulcer	+ +	+ +	+ + +	+ + +
Involvement of aortic branches	+ a	(+)	+ + +	+ + +
Aortic wall visualization b	+	+ + +	+ + +	+ + +
Comprehensive aortic assessment	+	+ +	+ + +	+ + +
Functional data	+ + +	+ + +	+ +	+ + +
Overall diagnostic reliability	+	+ + (+)	+ + +	+ + +
*Advantages/disadvantages* :				
Ease of use	+ + +	+ +	+ ++	+ +
Portability	+ + +	+ + +	–	−
Rapidity	+ + +	+ +	+ + +	+
Performed at bedside	+ + +	+ + +	–	−
Serial examinations	+ +	+	+ + (+) c	+ + +
Cost	–	–	– –	- - -
Radiation	0	0	–– –	0
Nephrotoxicity	0	0	–– –	- -
Need of sedation	–	+ + +	–	−

Notes: Modified from Erbel et al. 1

+ means a positive remark and – means a negative remark. The number of signs indicates the estimated potential value.

a Can be improved when combined with vascular ultrasound (carotid, subclavian, vertebral, celiac, mesenteric and renal arteries).

b PET can be used to visualize suspected aortic inflammatory disease.

c + + + only for follow-up after aortic stenting (metallic struts), otherwise limit radiation.

### Transthoracic/Transesophageal Echocardiography

Transthoracic echocardiography (TTE) is routinely used in the emergency scenario for differential diagnosis of many cardiologic conditions. In fact, it rapidly detects dissection-related complications, such as pericardial effusion with or without hemodynamic compromise, aortic regurgitation, and global or regional wall motion abnormalities, suggesting heart failure and/or acute coronary syndrome, respectively. However, while TTE allows accurate assessment of the aortic valve and ascending aorta, it often fails to comprehensively visualize the aortic arch and descending aorta (overall low accuracy). Furthermore, it is limited in presence of particular chest configuration, obesity, and pulmonary emphysema. On the other hand, TEE provided prompt availability and local expertise and can be performed to accurately evaluate patients with known or suspected AAS at bedside and/or in the operating room without the need for radiocontrast agents. Reverberation artifacts, suboptimal resolution for the distal ascending aorta/proximal aortic arch (TEE “blind spot” due to tracheal air shadowing), as well as for the abdominal aorta (distance from the imaging probe), underscore the need for a second imaging test (CT in the large majority of cases) in some patients. In addition, contraindications to TEE, such as esophageal disease and cervical spine disorders, should also be considered. Finally, TEE is less suited than CT and MRI for long-term imaging surveillance which requires a comprehensive assessment of the aorta and its branch vessels at easily identifiable landmarks. Another advantage is that serial measurements with high spatial orientation are easily possible.

### Transesophageal Echocardiography Studies

**Fig. 1:**
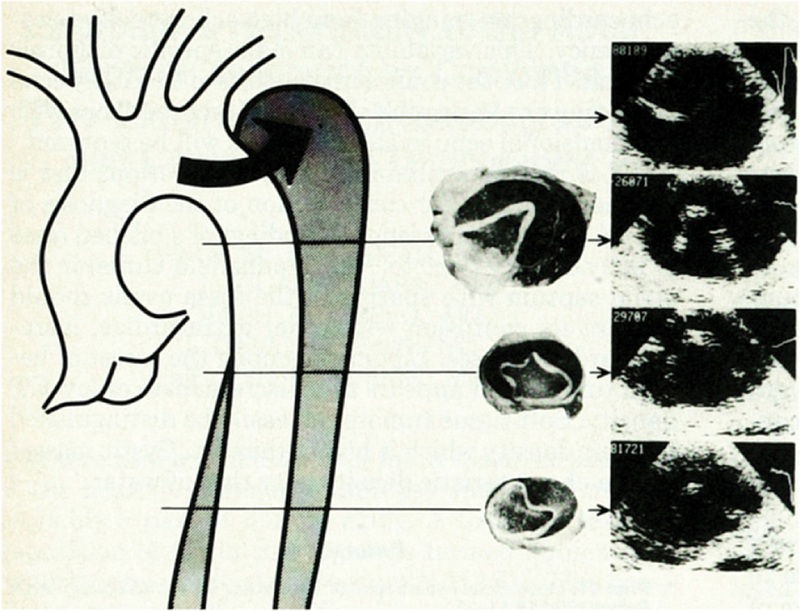
Anatomopathological sections and sonographic cross-sectional scans of the dissected descending thoracic aorta (Type III dissection), which revealed the similarity between transesophageal echocardiography and autopsy. Image courtesy: Börner at al. 10

The first description of AAD by TEE was reported by Börner et al 10 in 1984 ( Fig. 1 ). It took many years to convince that TEE is the method of choice in the acute setting, to show that it is safe and very accurate so that therapeutic decision-making is possible, and can be a new field for cardiologists during that time. Subsequently, in 1987, the accuracy of TEE for the diagnosis of AAD was compared with TTE, CT, and aortography in 21 patients undergoing TEE, confirming AAD diagnosis in all 21 patients examined. TTE accuracy was significantly higher in DeBakey Type I and II, compared with Type-III AAD, but this limitation could be overcome by TEE. In all patients, TEE was able to visualize the entire descending aorta, part of the aortic arch (due to interposition of trachea) and aortic root; additionally, it could identify, in all cases, the entry tear of intimal flap as compared with CT. 11

Already in this study, the authors pointed out a very important drawback, the ultrasound artifacts suggesting an aortic intimal flap due to a reverberation of the sound waves. verified by intraoperative controls in aortic aneurysms. 11 Furthermore, TEE could visualize underlying aorta and aortic valve morphologies predisposing to AAD, thus guiding additional treatment on the aortic valve complex. A new window to the heart was opened. 31

In a first European multicenter study, the accuracy of TTE/TEE was compared with CT (at that time only single detector systems) and aortography (MRI not yet available for emergency cases). TTE/TEE showed an excellent sensitivity and specificity (99 and 98%, respectively) with optimal positive and negative predictive value (98 and 99%, respectively), as compared with that of CT (83 and 100%, respectively, with positive and negative predictive values of 100 and 86%). In contrast, aortography showed a lower sensitivity (88%), specificity (94%), positive (96%), and negative (84%) predictive values but was crucial for identifying branch vessel involvement by dissection flap. 12 Characterization of AAD morphology and entry localization by TEE had also important prognostic implications. Patients with Type-III AAD with retrograde propagation of dissection to the ascending aorta represent a subgroup with poor prognosis, similarly to patients with high communicating AAD, as free communication between the true and false lumen (TL and FL, respectively) and high flow rates are associated with high pressure and wall stress in FL.

**Fig. 2:**
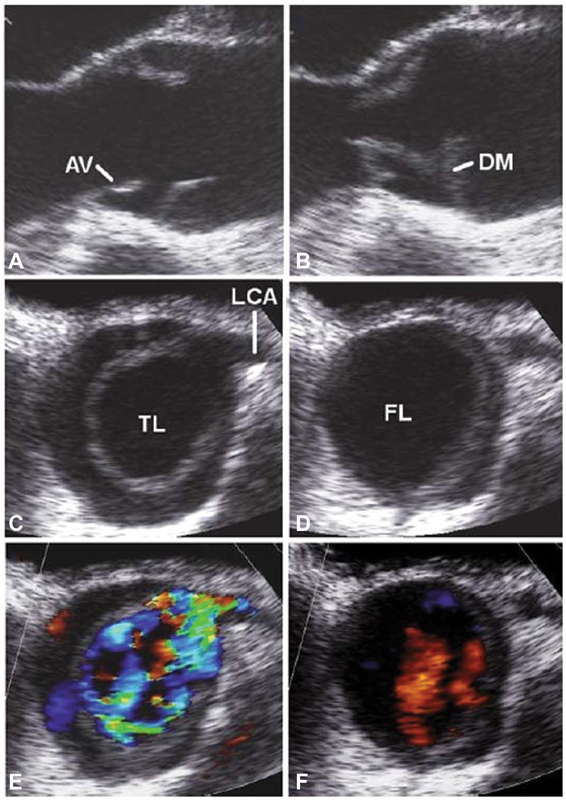
Intraoperative transesophageal echocardiography. Systolic ( **A, C, E** ) and diastolic ( **B, D, F** ) movement of the dissection membrane (DM) with prolapse of the DM through the aortic valve (AV). ( **C–F** ) Short-axis view of the AV showing the intermittent obstruction of the left coronary ostia (LCA). FL, false lumen; TL, true lumen. Image courtesy: Jánosi at al. 33

On the other hand, thrombus formation in the FL was a good prognostic factor; notably, signs of aortic rupture (mediastinal hematoma and pericardial and pleural effusion) could be visualized by TEE with high sensitivity and were associated with high mortality, independently of AAD type (51–75%). 32 Furthermore, TEE can provide information on flow dynamics between TL and FL in AAD patients over time. In a follow-up study involving 18 AAD patients, TEE was able to detect additional intimal tears which were not evident during the index TEE, as well as to distinguish between a biphasic flow from TL to FL with diastolic flow reversal (associated with large tears and no or localized thrombus), and a slowly circulating flow pattern (associated with small tears and extensive or progressive from distal to proximal thrombus formation) similar to that of spontaneous echocardiographic contrast. These findings may be helpful for early detection of complications leading to secondary surgery or to document the healing process. 9 Using electrocardiography (ECG) triggering, malperfusion of coronary arteries due to the obliteration by an intimal flap in diastole during reversed flow from the aorta to the coronary arteries could be visualized ( Fig. 2 ). 33 Combined with contrast enhancement, the value of echocardiography is further enhanced. 34

### Intracardiac Echocardiography

A study including 23 Type-B AAD patients compared the diagnostic performance of intracardiac echocardiography (ICE; intraluminal phased-array imaging [IPAI]), TEE, angiography, and IVUS. 17 IPAI was superior to TEE in detecting entries in the thoracic descending aorta. Furthermore, ICE correctly depicted the abdominal branches and their origins similarly to IVUS but on top of that, it was capable of showing flow in the abdominal side branches, thus proving that they were functionally not impaired. Notably, in patients with TL collapse, this technology allowed guidance of emergency fenestration. 17

### Intravascular Ultrasound

IVUS provides real-time imaging of aortic pathology and is a reliable and safe tool to guide stent–graft positioning. Since its first application in the 1990s in a patient with AAD, 18 IVUS imaging has been used as an enhanced technique for diagnostic and therapeutic procedures in patients with AAD. Weintraub and colleagues 18 validated the diagnostic performance of this technology in 23 patients with AAD, as compared with aortography, CT, and TEE. In all patients, IVUS was able to detect the intimal flap, TL and FL, involvement of branch vessels, presence of IMH, and thrombus formation. Notably, IVUS was superior to aortography, CT, and TEE in demonstrating the distal extent of dissection in ambiguous cases. In contrast, TEE depicted more clearly the dissection in the ascending aorta, potential multiple communications between TL and FL, and impaired flow in the coronary arteries. 17 In comparison to MRI, IVUS images demonstrated a surprising good agreement even for detection of PAU. 35 Furthermore, IVUS enabled real-time aortic diameter assessment and may be therefore useful in guiding stent–graft size. In a study involving 45 Type-B AAD patients, IVUS-assisted sizing yielded a greater increase in TL and reduction of FL and total aortic diameter during follow-up, compared with CT-assisted sizing ( Supplementary Fig. S2 ; available in the online version). 19 Although IVUS cannot be recommended routinely due to its cost inefficiency, it should be taken into account as a resourceful tool when CT image quality is poor and, in patients, with hemodynamic compromise. In this setting, poor volume filling at the time of CT may lead to underestimation of aortic diameter. In contrast, IVUS enables the measurement of the exact diameter quite before thoracic endovascular aortic repair (TEVAR) after stabilization of the patient with volume filling and reduces the incidence of stent–graft oversizing. 20 36

However, pitfalls of IVUS are to be remembered. If the common IVUS catheter with rotating transducers is used, flow information is lacking, as Doppler is not possible. The catheter tip follows the direction of the guidewire.

The dedicated ICE catheters are opening another view, because the aorta can be imaged from the superior and inferior cava, but also from the heart, right ventricle. 37 The full flexibility of the catheter tip in three dimensions is a great advantage. In addition, the ICE catheter can be moved within the aorta which is helpful to image the entry tears or ostium of the side branches. Meanwhile, IVUS had been used to guide interventions. In the no-reflow situation, fenestration had been the only approach which could be used in the cath laboratory. 38 39 Now, in the era of TEVAR/endovascular aneurysm repair (EVAR), the indication for a fenestration is rare but an excellent solution when a no reflow persists after stenting. Particularly for biopsy of a mass of unknown etiology within the lumen of the aorta, the support of imaging is extremely helpful. 40

Although the correlation between aortic diameters at CT and IVUS was overall good along the aorta (especially abdominal aorta), IVUS tended to overestimate luminal diameter due the tortuosity of the aortic arch and the drift out of the coaxial axis. The greatest difference in diameter measurements was observed at the origin of the left subclavian artery (IVUS – CT = 2.69 ± 2.03 mm), a common proximal landing zone in TEVAR ( Supplementary Fig. S2 ; available in the online version). 19

Therefore, one possible strategy for stent–graft selection is to use only the minimum measured diameter when you are aware of an off-center measurement of IVUS. 19

IVUS is very helpful for the surgeon when problems exist related to the abdominal and thoracic aorta, because the passage of guidewires and catheters can be guided.

### Computed Tomography

Given the excellent accuracy, the short-time image acquisition/processing, widespread availability, and patient tolerance, CT is considered to be the ideal diagnostic tool in emergency scenarios. 1 Furthermore, it enables a complete and detailed map of the entire aorta and its branches with high spatial/contrast resolution without limiting for image window. In the beginning, only single-row detectors were available but nowadays, dual source scanners, and up to 624-scan lines are present. The resolution increased significantly combined with rapid data acquisition. Nonenhanced CT followed by contrast-enhanced CT should be preferred to assess patients with suspected AAS. However, ECG gating is recommended to avoid artifacts. The main limitations are related to lack of aortic regurgitation detection (often present in Type-A AAD) and lack of measurement of flow and flow direction. Furthermore, the impossibility to be performed at bedside limits its feasibility in unstable patients. CT disadvantages consist in the increased risk of allergic reactions due to the use of iodinated contrast agents and iodinated contrast medium. Cumulative exposure to radiation remains a major drawback (especially in young patients) during surveillance where serial CT scans are needed.

Rylski et al 22 investigated changes in descending aortic geometry due to dissection among 25 Type-B AAD patients. All the patients underwent CT less than 2 years before AAD and immediately after. The largest increase in postdissection diameter was registered at the level of the middescending thoracic aorta (+6.4 mm; +23%). In addition, an increase in length and volume of the descending thoracic aorta was noted. Interestingly, the investigators found that the predissection aortic diameter appears to be the most similar to the postdissection maximum diameter of the TL (+2.5 mm).

### Magnetic Resonance Imaging

The value of MRI in aortic disease was a main topic of the study by Nienaber et al 41 42 in Hamburg taking into account that MRI has limited value in the acute setting but great advantages in the chronic phase. In addition, different types of aortic disease were visualized similar to TEE scans but for the whole aorta. Very early, the Mainz group pointed to the diagnostic value of MRI in aortic disease. 43 The main emphasis was found in the differential diagnosis of aortic disease. IMH attracted the interest, as it became clear that this type of aorta pathology quite often leads to full aortic dissection and is often found when aortic dissection was suggested, with the advantage of imaging the whole aorta and not only the thoracic part with high resolution. 44 45 However, IMH had been described by pathologists quite early 46 47 as aortic dissection, without intimal rupture, diagnosed with MRI and CT. 48 Nevertheless, problems exist for the detection and localization of calcification and metal stents. The ability to detect flow in TL and FL, to detect the perfusion direction and the degree of flow are very important, as well as the optimal spatial orientation. Therefore, the first attempts were done in Essen by Eggebrecht et al 49 to use MRI for stent implantation ( Supplementary Fig. S3 ; available in the online version). Further refinement of real-time MRI will provide an option for enhanced stent graft implantation in the future.

### Positron Emission Tomography/Computed Tomography

^18^ F-FDG PET/CT is an imaging technique that provides morphological and metabolic information by detecting increased FDG uptake. Provided the proven role of this imaging modality in the field of oncology and patients with vasculitis, PET/CT imaging may play also an important prognostic role in patients with AAS. In 60 patients with Type-B AAS, a pathological FDG uptake in the aortic wall was associated with a significant increase in inflammatory biomarkers (i.e., C-reactive protein and D-dimer). Although PET positivity did not affect in-hospital outcome, during follow-up, PET-positive patients had a greater risk of disease progression, aorta-related mortality, and reintervention than PET-negative patients. Interestingly, the combination of PET results with D-dimer levels had the best discriminant value of major adverse events, compared with PET, D-dimer, and C-reactive protein taken individually ( Supplementary Fig. S4 ; available in the online version). 24 Thus, this combined strategy (metabolic and biochemical information) may prove to be helpful in more accurately identifying patients with multiple risk factors (amount of thrombus, active FL, inflammation in the aortic wall, intimal tears, etc.) who are at higher risk for disease progression and complications during follow-up. Similarly, evidence of a PET-positive aortic pathology may justify a closer follow-up or a more aggressive treatment with surgery/endovascular therapy in medically treated AAD patients to improve the outcomes. 23 24

## Role of Biomarkers in Acute Aortic Syndromes

As in other cardiovascular diseases, biomarkers represent an important part of the comprehensive assessment of patients with suspected or overt AAS ( Table 6 ). Key pioneer studies undertaken by Mainz–Essen investigators have shed the light on the diagnostic and prognostic role of biomarkers.

**Table 6:** Laboratory tests required for patients with acute aortic syndromes

Laboratory tests	To detect signs of:
Red blood cell count/hemoglobin	Blood loss, bleeding, anemia
White blood cell count	Infection, inflammation (systemic inflammatory response syndrome [SIRS])
C-reactive protein	Inflammatory response
Interleukin-6	Vascular inflammation
Procalcitonin	Differential diagnosis between SIRS and sepsis
Creatine kinase	Reperfusion injury, rhabdomyolysis
Troponin I or T	Myocardial ischemia, myocardial infarction
D-dimer	Aortic dissection, pulmonary embolism, thrombosis
Creatinine	Renal failure (existing or developing)
Alanine aminotransferase/aspartate transaminase	Liver ischemia, liver disease
Lactate	Bowel ischemia, metabolic disorder
Glucose	Diabetes mellitus
Blood gases	Metabolic disorder, oxygenation
Brain natriuretic peptide/N-terminal probrain natriuretic peptide	Heart failure

Note: Modified from Erbel et al. 1

### D-Dimer as a Diagnostic Marker of Acute Aortic Dissection

**Fig. 3:**
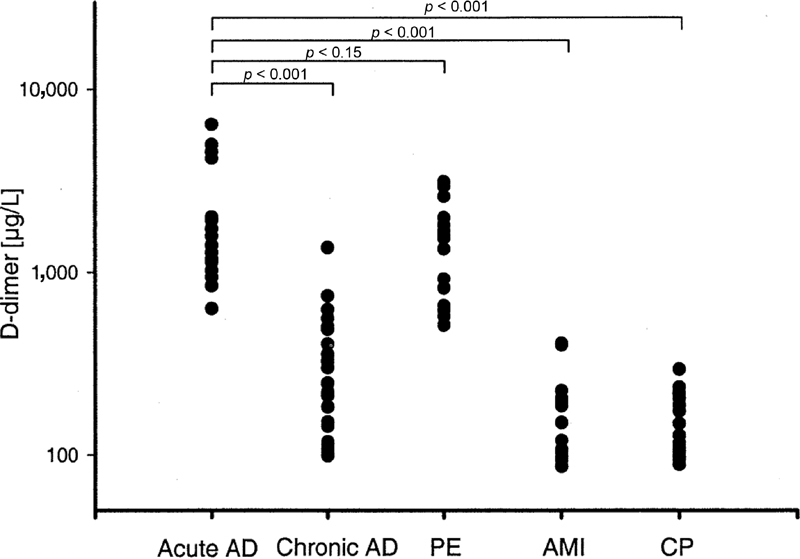
Comparison of D-dimer values between the different patient groups ( *p* -values adjusted according to Bonferroni correction). AD, aortic dissection; AMI, acute myocardial infarction; CP, chest pain; PE, pulmonary embolism. Image courtesy: Eggebrecht et al. 25

In 2004, a multicenter study involving 64 chest pain patients showed that D-dimer was highly elevated in those with AAD, with similar levels to pulmonary embolism and significantly higher than those with acute myocardial infarction, chronic aortic dissection, and other causes of noncardiac chest pain ( Fig. 3 ). 25 Additionally, a systemic inflammatory response including significant increase of white blood cells, C-reactive protein, and fibrinogen was evident in AAD patients. 25 26 These results were furtherly validated in 2017 with a single-center study involving 522 patients, 231 of them suffering from AAS (159 AAD, 35 IMH, and 37 PAU). 27 Among AAS patients, those with AAD and IMH had comparably increased D-dimer levels, as compared with patients with pulmonary embolism, myocardial infarction, and other causes of chest pain, whereas this was not the case of PAU patients. Similarly, the discriminant value of D-dimer was excellent both for AAD and IMH (area under the curve [AUC] = 0.96, sensitivity = 99%, and specificity = 67%; and AUC = 0.98, sensitivity = 100%, and specificity = 67%, respectively) but not for PAU (AUC = 0.69, sensitivity = 64%, and specificity = 67%). 27

### Integrated Clinical Biomarkers and Imaging Diagnostic Algorithm

In 2010, the American and ACC/AHA guidelines for the diagnosis and management of patients with thoracic aortic disease designed an initial a priori risk estimate based on predisposing conditions, pain features and clinical examination to identify patients at low (score: 0–1) and high (score: 2–3) risk for AAD ( Supplementary Fig. S5A ; available in the online version). 9 The proposed risk estimate tested among 2,538 patients enrolled in International Registry of Acute Aortic Dissection (IRAD) registry showed an excellent diagnostic sensitivity (95.7%). 50 Subsequently, the 2014 European Society of Cardiology (ESC) guidelines 1 on the diagnosis and treatment of aortic diseases integrated the pretest probability estimation with laboratory (D-dimer) and imaging tests (echocardiography and/or CT) to rapidly confirm or rule out the diagnosis of aortic dissection ( Supplementary Fig. S5B ; available in the online version). 1 Validation into the real-world management of patients with suspected AAD of the ESC guidelines integrated diagnostic algorithm was performed among 376 chest pain patients, 85 of them with AAS. 28

A “high probability” aortic dissection detection (ADD) score (2 or 3) detected AAS with good specificity (98.9%) with a failure rate of 9.7%. A “low probability” ADD score (0 or 1) combined with negative D-dimer (<0.5 ng/L) safely and efficiently ruled out AAS with a negative predictive value of 98.9% and a low failure rate (1.1%). 28 These findings were confirmed also by another group in a prospective multicenter study. 51 Therefore, whereas in patients with “high probability,” ADD risk score D-dimer assessment is not necessary and expedite aortic imaging (CT or TEE) should be warranted to confirm AAD, it plays a pivotal role when assessed in individuals with a “low probability” ADD risk score. First, a negative D-dimer rules out AAD, owing to the excellent negative predictive value; and second, a positive D-dimer warrants further aortic imaging which may lead to the detection of AAS in patients with an atypical presentation that would remain undetected on the basis of the ADD risk score alone. However, it has to be underlined that D-dimer may result within normal limits in the case of AAD with thrombosed FL, IMH, and PAU.

### Hemoglobin

Gorla et al 30 investigated among 144 Type-B AAS undergoing TEVAR, the prognostic impact of preoperative anemia and postoperative hemoglobin (Hb) drop on in-hospital mortality, and the incidence of acute kidney injury (AKI). Three groups of patients were identified (no/mild, moderate, and severe) based on values of preoperative anemia and postoperative Hb drop. Data showed that postoperative AKI was higher in the severe and moderate anemia groups than in the no/mild anemia group and that in-hospital mortality and AKI were higher in patients with severe postoperative Hb drop than in patients with moderate or mild postoperative Hb drop. 30

Therefore, preoperative anemia and postoperative Hb drop appear to predict in-hospital mortality and to be significant risk factors for AKI. 30

## Conclusion

The unique 35-year-long Mainz–Essen experience represents a milestone of the aortic disease research journey. New horizons have been opened on biomarker-imaging interplay to provide timely diagnosis, assessment of prognosis, and guidance for therapeutic interventions. In this regard, the development and subsequent validation of 2014 ESC guidelines on the diagnosis and treatment of aortic diseases diagnostic algorithm represents a milestone on the evaluation of patients with suspected AAS in the emergency scenario. However, more should be done to investigate potential serological and imaging biomarkers signaling clinically silent cases of early anatomopathological changes of the aortic wall to implement optimal preventive measures. On the other hand, there is a need to prevent redissection or aneurysm formation after optimal interventions of the acute index event. Thus, the primary and secondary aortic disease prevention landscape remains to be explored in the coming years. In this scenario, genetics, proteomics, biomarkers, and advances in imaging may play a major role in an integrated multiparametric approach.

## Notes

Editor's Commentary
These two articles, Part I (published here) and Part II (to be published in the next issue of *AORTA* ), summarize clearly the advances in thoracic aortic disease—imaging, diagnosis, and treatment—made over the last several decades. Many of these advances were originated by the Essen team, who authored these articles. I hope our readers find the narratives and the tabulations of relevant key articles extremely useful as historical, clinical, and didactic tools. These articles are reflective of great concentration, innovation, and creativity in treatment of aortic diseases.
