# Supplementary material for: Initiating a New Era of Cardiovascular Diagnosis and Therapy in Acute Aortic Syndromes: The Mainz–Essen Experience (Part I)—Imaging and Biomarkers
Source: Aorta (Stamford). 2021 Nov 8;9(4):127–38. doi: 10.1055/s-0041-1730295 (PMC8642076; doi:10.1055/s-0041-1730295)
Supplement: Supplementary file 1 — Supplementary Material [file 10-1055-s-0041-1730295-s200030.pdf]

Supplementary Box S1 Review protocol and search strategy

Review protocol
A protocol for this review was prospectively developed, detailing specific objectives and criteria for study selection. For the study purpose, the Mainz-Essen experience on acute aortic syndrome (AAS) was summarized by considering original articles from single-center or multicenter studies performed at West German Heart Centre, Essen, Germany or at the cardiovascular department of Johannes Gutenberg University, Mainz, Germany. Articles in German language, case reports, editorials and reviews were excluded
Search strategy
An electronic MEDLINE search was performed combining the search terms “Erbel R” or “Jakob H” (former chief cardiologist and chief heart surgeon, respectively, at West German Heart Centre in Essen) with “aortic disease,” “aortic dissection,” “intramural hematoma,” “penetrating aortic ulcer,” and “transection.” This search included studies published until December 2018. The search was supplemented by manually reviewing the reference list of the retrieved review articles. A flowchart of the study selection process is shown in ►**Supplementary Fig. S1**
Studies on the role of different imaging modalities in AAS
The search retrieved 14 studies (8 single center and 6 multicenter); of them, 6 were focused on transthoracic echocardiography/transesophageal echocardiography,[11–16] 1 on intracardiac echocardiography,[17] 4 on intravascular ultrasound,[18–21] and 1 on computed tomography (CT),[22] and 2 on positron emission tomography/CT[23,24] (►**Table 3**)
Studies on the role of biomarkers in AAS
The search retrieved 6 studies (5 single-center and 1 multicenter); of them, 5 were focused on D-dimer,[25–29] and 1 on hemoglobin/creatinine[30] (►**Table 4**)

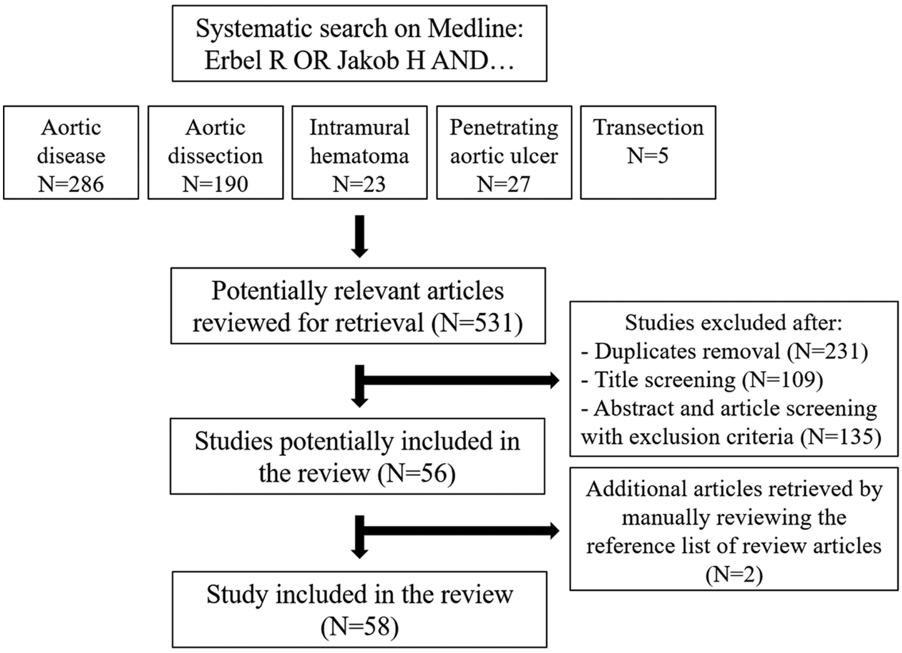

Supplementary Fig. S1 Study flowchart.

 © 2021. The Author(s).

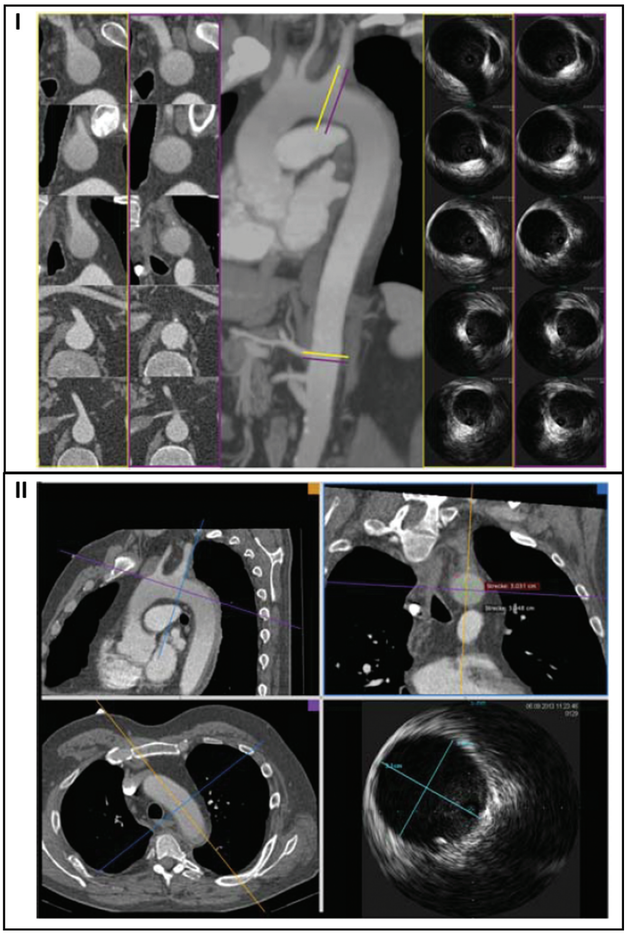

Supplementary Fig. S2 Computed tomography (CT) and intravascular ultrasound (IVUS) performance in intramural hematoma detection. (Part I): CT and IVUS mapping of the aortic arch and descending aorta: to obtain comparable measurements by IVUS and CT angiography the aortic root and the aortic branches (yellow line) were used as anatomical references. In CT measurements of the aortic arch, reconstructions were made in addition to the axial slides. The first most circular shape at the end of the branch was selected for measurement (purple line). (Part II): Measurement of the aortic lumen at the left subclavian artery in a patient with an intramural hematoma. Diameters in the aortic arch were measured after three-plane reconstruction, taking the transversal sections perpendicular to the central line of the aortic lumen. Image courtesy: Jánosi RA, et al.[19]

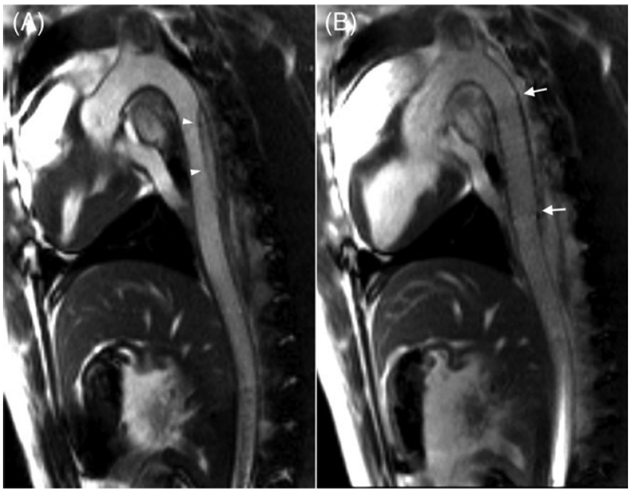

Supplementary Fig. S3 (**A**) Preinterventional high-resolution magnetic resonance imaging (MRI) in parasagittal orientation showing the dissection flap (arrowheads) in the proximal descending thoracic aorta. (**B**) Corresponding postinterventional MRI demonstrating correct position of the stent–graft (arrows) with complete coverage of the dissection. Image courtesy: Eggebrecht et al.[49]

 © 2021. The Author(s).

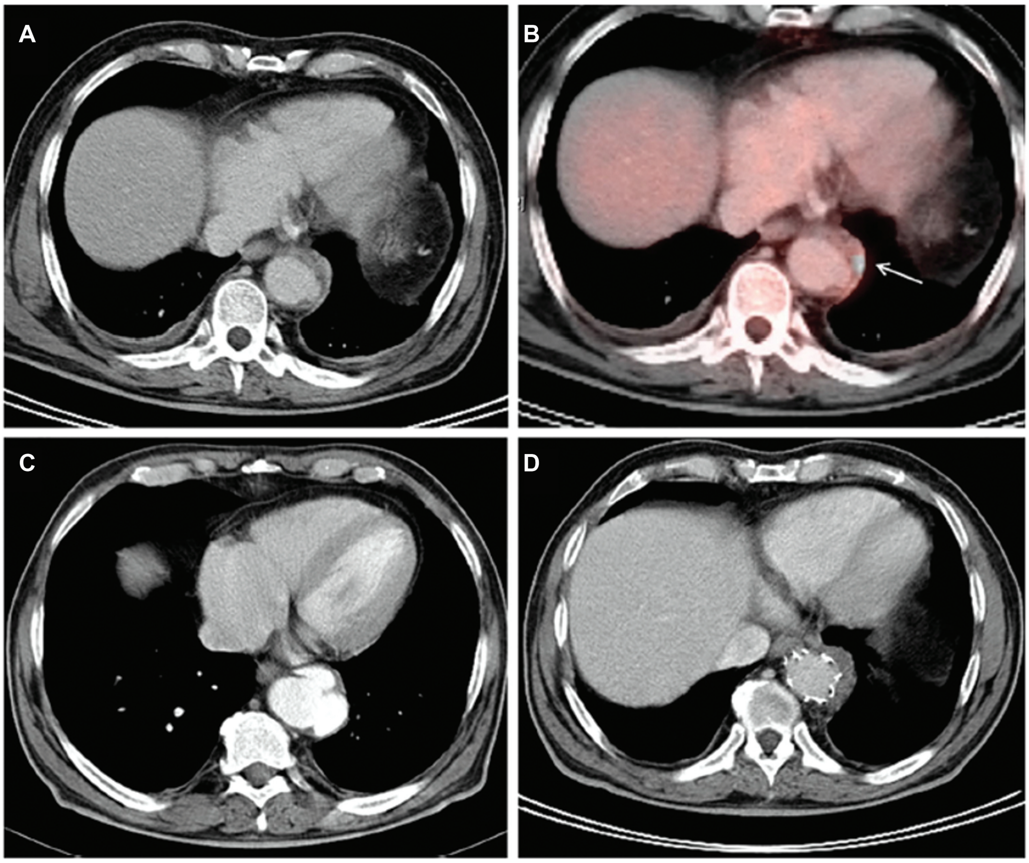

Supplementary Fig. S4 Positron emission tomography/computed tomography (PET/CT) images. A 79-year-old male patient was admitted to our department because of chest pain, fever and positive blood cultures. PET/CT imaging shows a penetrating aortic ulcer associated with intramural hematoma of the descending aorta (**A**) and increased ^{18}F-fluorodeoxyglucose uptake in the aortic wall (arrow); (**B**) the patient was treated conservatively with antihypertensive agents and antibiotics. CT performed 2 months later shows the development of an aortic pseudoaneurysm; (**C**) CT after stent-graft implantation shows the complete exclusion of the pseudoaneurysm (**D**). Image courtesy: Gorla et al.[24]

 © 2021. The Author(s).

A

High-risk conditions	High-risk pain features	High-risk examination features
• Marfan syndrome<br>(or other connective tissue diseases)<br>• Family history of aortic disease<br>• Known aortic valve disease<br>• Known thoracic aortic aneurysm<br>• Previous aortic manipulation (including cardiac surgery)	• Chest, back, or abdominal pain described as any of the following:<br>- abrupt onset<br>- severe intensity<br>- ripping or tearing	• Evidence of perfusion deficit:<br>- pulse deficit<br>- systolic blood pressure difference<br>- focal neurological deficit (in conjunction with pain)<br>• Aortic diastolic murmur (new and with pain)<br>• Hypotension or shock

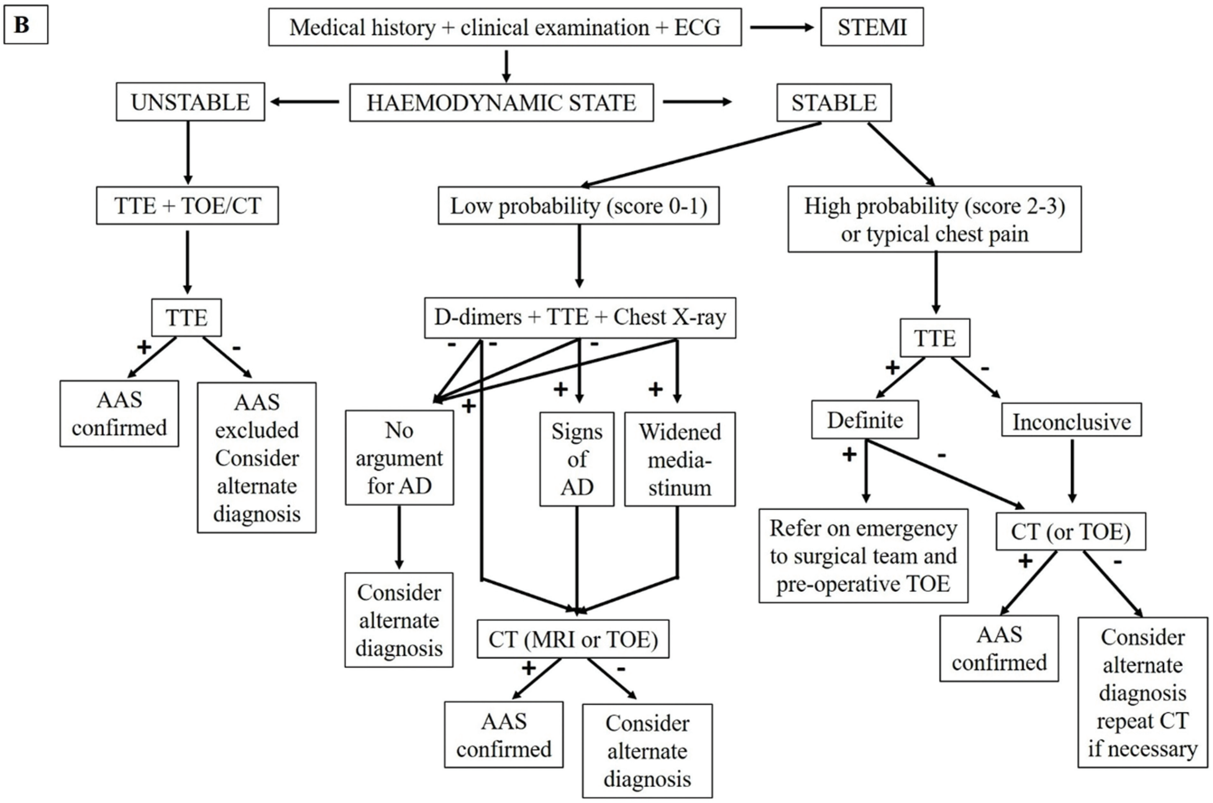

Supplementary Fig. S5 (**A**) Clinical data useful to assess the a priori probability of acute aortic syndrome (AAS); (**B**) Flowchart for decision-making based on pretest sensitivity of AAS. AD, aortic dissection; CT, computed tomography; ECG, electrocardiography; MRI, magnetic resonance imaging; STEMI, ST-elevation myocardial infarction; TEE/TOE, transesophageal echocardiography; TTE, transthoracic echocardiography. Image courtesy: Erbel et al.[1,9]

 © 2021. The Author(s).
